# A Case Report on Internal Hernia as an Unusual Cause of Small Bowel Obstruction: A Diagnostic Triumph Unveiled by Computed Tomography Scan

**DOI:** 10.7759/cureus.55526

**Published:** 2024-03-04

**Authors:** Pavithra Devi, Anand Hatgaonkar, Viram Tanksale, Vaishali Dhawan, Akhilesh Kamble, Meet Jobanputra

**Affiliations:** 1 Radiodiagnosis, Datta Meghe Medical College, Datta Meghe Institute of Higher Education and Research (Deemed to be University), Wardha, IND; 2 Radiodiagnosis, LGI Hospitals, Nagpur, IND; 3 Radiology, Jawaharlal Nehru Medical College, Datta Meghe Institute of Higher Education and Research (Deemed to be University), Wardha, IND; 4 General Surgery, Datta Meghe Medical College, Datta Meghe Institute of Higher Education and Research (Deemed to be University), Wardha, IND

**Keywords:** surgical repair, computed tomography, acute small bowel obstruction, paraduodenal hernia, internal hernia

## Abstract

Paraduodenal hernia is a type of internal hernia caused by defects in the peritoneum during fetal development. It is one of the uncommon causes of intestinal obstruction; diagnosing it and intervening promptly are required. In this case report, we describe our experience treating an adult male patient who presented with symptoms of acute small bowel obstruction and was later diagnosed with internal hernia on further evaluation. The purpose of this case report is to demonstrate the importance of imaging modalities, particularly computed tomography (CT) scan, in diagnosing these cases. Because of their mysterious symptoms, paraduodenal hernias are usually diagnosed late or incidentally. Although they are uncommon, they are far more likely to cause bowel obstruction and strangulation. Immediate surgical intervention is required following the diagnosis.

## Introduction

An uncommon etiology of intestinal obstruction is attributable to internal hernia, representing an incidence of approximately 0.6-5.8% [1]. Within the spectrum of internal hernias, the left paraduodenal hernia (PDH) prevails as the most frequently encountered, followed by the right PDH. PDHs, constituting approximately 53% of all internal hernia cases, stand out as the predominant subtype [2]. The diagnosis of PDHs poses a challenge owing to their infrequent occurrence and the presence of nonspecific symptoms [3]. Accurate clinical identification proves intricate in the absence of distinctive symptoms or discernible clinical findings. Given the potential complications, such as bowel obstruction or ischemia, prompt diagnosis assumes paramount importance. A heightened level of suspicion becomes imperative, and the utilization of computed tomography (CT) scans emerges as a pivotal tool in corroborating the diagnosis [4]. This case report elucidates the imaging findings in an adult male patient diagnosed with PDH, underscoring the indispensable role of radiological investigations in confirming the diagnosis.

## Case presentation

A 45-year-old male sought medical attention at the emergency department of a tertiary care hospital in central India, reporting symptoms of abdominal pain, distension, and vomiting for two days.

Clinical progression

The patient experienced intermittent crampy abdominal pain, accompanied by episodes of vomiting and abdominal distension. Upon physical examination, generalized discomfort was noted, without palpable mass or signs indicative of peritoneal irritation. Subsequently, the patient underwent an erect abdominal radiograph and an abdominal ultrasound.

Diagnostic evaluation

**Figure 1:**
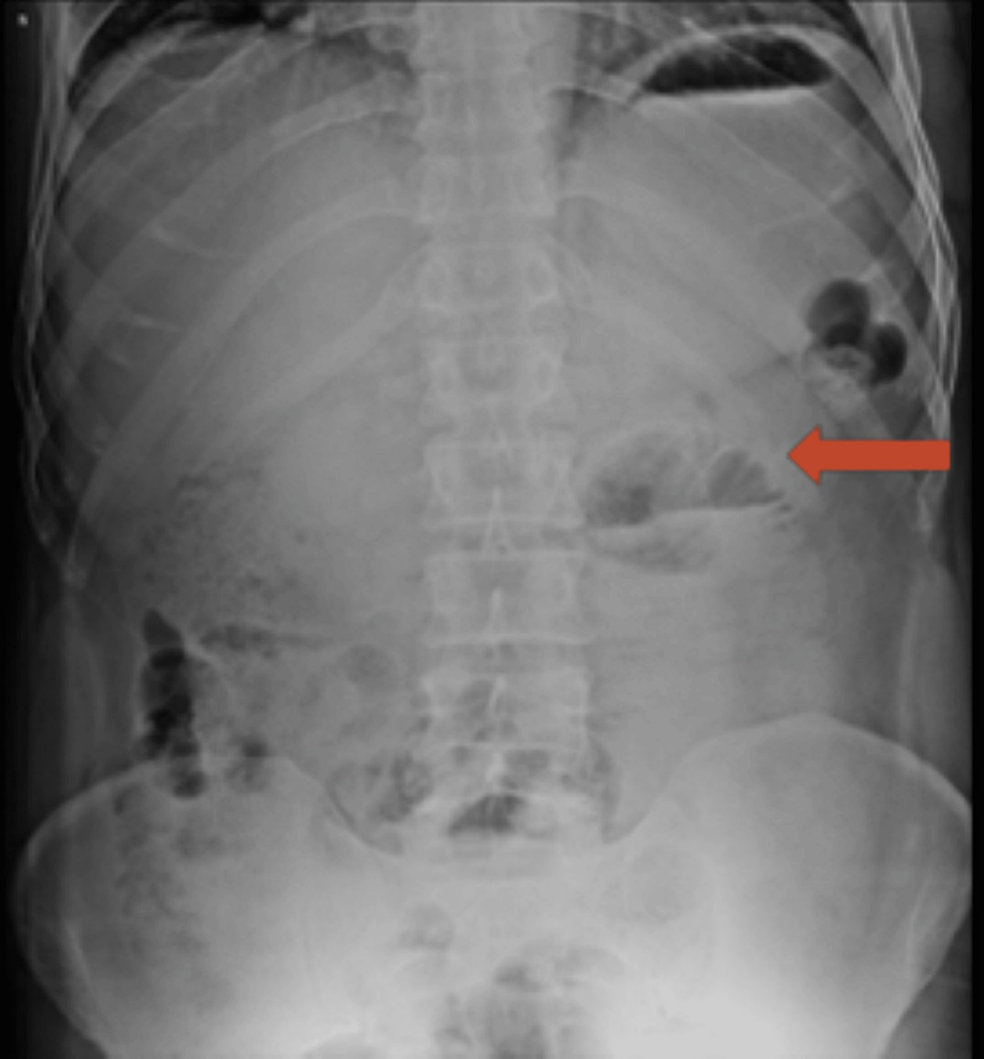
X-ray abdomen erect view. Few air-fluid levels (red arrow) noted in the central abdomen and pelvic region with dilated small bowel loops.

The erect abdominal radiograph revealed abnormal air-fluid levels in the mid abdomen, along with dilated small bowel loops, suggesting the presence of small bowel obstruction (Figure 1). The abdominal ultrasound showed dilated small bowel loops in the central abdomen, without any discernible transition point or mass. 

**Figure 2:**
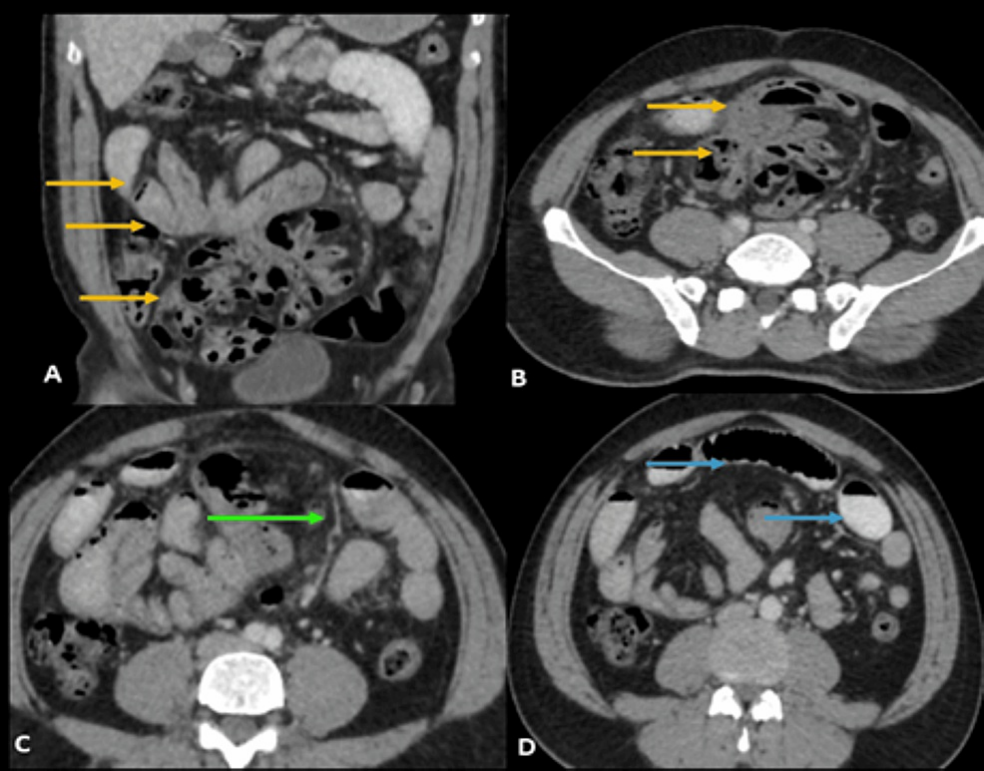
Axial and coronal contrast-enhanced CT images. (A,B) Abnormal sac-like structure with jejunal and ileal loops in the umbilical and hypogastric regions (yellow arrows). (C) Superior mesenteric vessels stretching along the medial aspect of the sac of bowel loops (green arrow). (D) Dilated small bowels adjacent to the sac (blue arrows). CT: computed tomography.

Further imaging via abdominal CT, utilizing oral and intravenous contrast, disclosed a sac-like structure containing clusters of jejunal and ileal loops in the umbilical and hypogastric regions (Figure 2A, B). The superior mesenteric vessels were observed along the anteromedial aspect of the bowel loop sac (Figure 2C). Multiple air-fluid levels were evident within the sac with adjacent dilated small bowels having a maximum diameter of up to 3.1 cm, suggestive of intestinal obstruction (Figure 2D). Multiplanar reconstruction emphasized the distinctive stretching of mesenteric vessels, reinforcing the relationship of the loops.

Contrast-enhanced imaging facilitated the identification of vascular relations in the hernia, assessment of bowel wall attenuation for viability, and determination of the necessity for surgical intervention. The diagnosis of right PDH was confirmed based on CT findings, indicating clustered bowel loops, characteristic mesenteric vessel stretching, and localized herniation.

Intervention and outcome 

**Figure 3:**
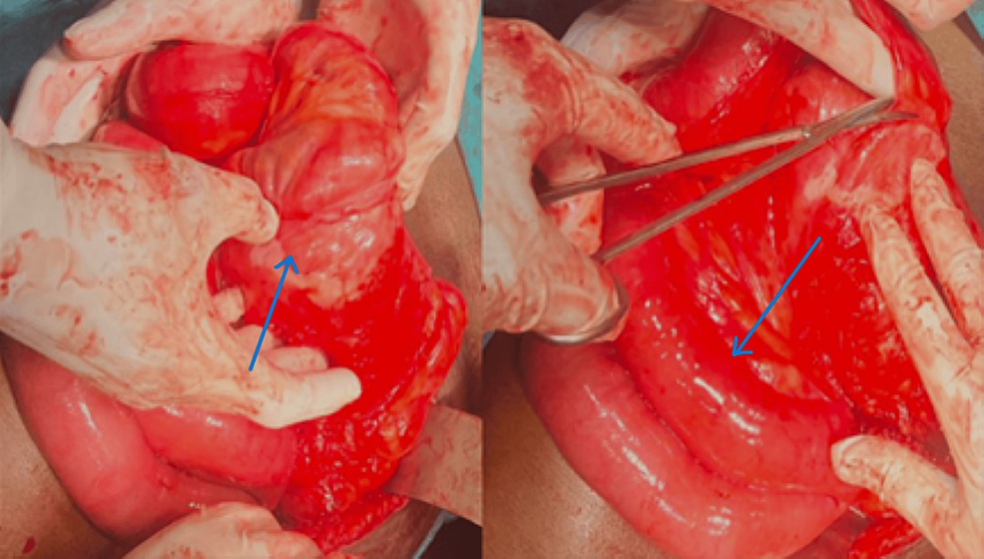
Intraoperative image. Findings aligned with the imaging features showing sac of bowel loops with adjacent dilated bowel loops.

The treating surgeon was promptly informed of the imaging findings. Subsequently, the patient underwent an emergency open exploratory laparotomy to address the obstruction, confirm the diagnosis, and conduct surgical repair of the hernia. Intraoperative observations aligned with the imaging results, revealing associated peritoneal adhesions (Figure 3). The herniated bowel loops were successfully reduced, and the defect in the paraduodenal area was repaired.

## Discussion

An internal hernia is characterized by protrusion into peritoneal pouches or apertures, differing from hernias resulting from abnormalities in the abdominal walls. The presence of a sac containing an abdominal organ, whether acquired or congenital, defines the occurrence of internal hernias. While hernia symptoms may manifest at any age, they are less frequent in the initial two decades of life, with the median diagnostic age typically falling between the fourth and sixth decades [5].

Internal hernias, although uncommon, are a well-recognized cause of intestinal obstruction. The most prevalent type, constituting approximately 53% of cases, is the PDH, which exists in two forms: right-sided PDH and left-sided PDH [6]. The mesentericoparietal fossa of Waldeyer is the aperture on the right, while the fossa of Landzert is located on the left, which accounts for three times as many cases [7]. The fossa of Waldeyer results from the failure of the posterior parietal peritoneum to fuse with the ascending mesocolon, situated beneath the duodenum's third portion, with the superior mesenteric vessels running along the medial free margin [8].

Clinical manifestations are typically nonspecific, encompassing vague abdominal discomfort, distension, vomiting, and recurrent intestinal obstruction. Although barium studies have described findings of this condition, CT has become the gold standard in contemporary radiology practice [9]. CT reveals the variable location of herniated bowel loops, featuring signs of small bowel obstruction such as dilated bowel loops and air-fluid levels. Additionally, there may be associated stretching, engorgement, or torsion of mesenteric vessels. A distinguishing feature of right PDH is the proximity of the superior mesenteric artery and right colic vein to the anteromedial border of the encapsulated small bowel loops [4].

High-resolution, multiplanar images obtained through multislice CT play a crucial role in providing distinctive and demonstrative information, facilitating early and accurate diagnosis essential for surgical treatment planning. An emphasis should be placed on investigating radiographic indicators of its complications, such as intestinal ischemia and hypoperfusion, considering the significant overall mortality rate exceeding 50% [10]. The treatment strategy for PDHs involves hernia reduction combined with either defect correction or enlargement of the hernial orifice. Failure to resolve the obstruction may lead to serious consequences, including perforation and ischemia.

## Conclusions

This case report emphasizes the importance of utilizing CT scans in the diagnosis of PDHs. The identification of distinctive radiological features is imperative for achieving precise diagnostic results and facilitating timely surgical intervention, ultimately contributing to positive patient outcomes. Thus, the diagnosis of PDH poses a considerable challenge, necessitating a comprehensive understanding of peritoneal and mesenteric fold anatomy, coupled with a heightened level of clinical suspicion.
